# Community Analysis of Chronic Wound Bacteria Using 16S rRNA Gene-Based Pyrosequencing: Impact of Diabetes and Antibiotics on Chronic Wound Microbiota

**DOI:** 10.1371/journal.pone.0006462

**Published:** 2009-07-31

**Authors:** Lance B. Price, Cindy M. Liu, Johan H. Melendez, Yelena M. Frankel, David Engelthaler, Maliha Aziz, Jolene Bowers, Rogan Rattray, Jacques Ravel, Chris Kingsley, Paul S. Keim, Gerald S. Lazarus, Jonathan M. Zenilman

**Affiliations:** 1 Translational Genomics Research Institute, Flagstaff, Arizona, United States of America; 2 Northern Arizona University, Flagstaff, Arizona, United States of America; 3 Johns Hopkins Medical Institutions, Baltimore, Maryland, United States of America; 4 Institute for Genome Sciences, University of Maryland School of Medicine, Baltimore, Maryland, United States of America; Columbia University, United States of America

## Abstract

**Background:**

Bacterial colonization is hypothesized to play a pathogenic role in the non-healing state of chronic wounds. We characterized wound bacteria from a cohort of chronic wound patients using a 16S rRNA gene-based pyrosequencing approach and assessed the impact of diabetes and antibiotics on chronic wound microbiota.

**Methodology/Principal Findings:**

We prospectively enrolled 24 patients at a referral wound center in Baltimore, MD; sampled patients' wounds by curette; cultured samples under aerobic and anaerobic conditions; and pyrosequenced the 16S rRNA V3 hypervariable region. The 16S rRNA gene-based analyses revealed an average of 10 different bacterial families in wounds—approximately 4 times more than estimated by culture-based analyses. Fastidious anaerobic bacteria belonging to the Clostridiales family XI were among the most prevalent bacteria identified exclusively by 16S rRNA gene-based analyses. Community-scale analyses showed that wound microbiota from antibiotic treated patients were significantly different from untreated patients (*p* = 0.007) and were characterized by increased Pseudomonadaceae abundance. These analyses also revealed that antibiotic use was associated with decreased Streptococcaceae among diabetics and that Streptococcaceae was more abundant among diabetics as compared to non-diabetics.

**Conclusions/Significance:**

The 16S rRNA gene-based analyses revealed complex bacterial communities including anaerobic bacteria that may play causative roles in the non-healing state of some chronic wounds. Our data suggest that antimicrobial therapy alters community structure—reducing some bacteria while selecting for others.

## Introduction

Chronic wounds cause substantial morbidity and economic burden that is borne disproportionately by diabetic, geriatric, and immobilized patients [1]. Generally associated with venous, arterial, or metabolic abnormalities [2], more than 90% of the chronic wounds fall into three categories: diabetic ulcers, venous ulcers, and pressure ulcers [3]. Four processes have been hypothesized to be the underlying cause in chronic wounds: 1) local tissue hypoxia, 2) repetitive ischemia-reperfusion injury, 3) altered cellular and systemic stress response, and 4) bacterial colonization [3]. Among these, bacterial colonization is of particular interest to clinicians for its association with chronic wound infections and as targets for novel wound therapies.

Although bacterial colonization occurs in all chronic wounds, the differentiation between *wound colonization* and *invasive infection* is not well defined. The hypothesized impact of bacterial colonization on wound healing ranges from detrimental to beneficial depending on the colonizing bacterial species and relative load [4]. Wound colonization is typically polymicrobial in nature (i.e., consisting of multiple bacterial species) [5]; thus, broad-spectrum antibiotics may modify, but not eliminate bacterial colonization.

Clinicians routinely use antibiotics for chronic wound care, but their optimal use and benefit remain unclear [6]. Systematic reviews have found little evidence for the benefit of antibiotic therapy on wound healing [7], [8]. Yet, patients with chronic wounds continue to receive more antibiotic therapy than age- and sex-matched non-wound patients [9], even as antibiotic-resistant organisms such as methicillin-resistant *Staphylococcus aureus* and antibiotic-resistant *Pseudomonas* are becoming more prevalent in wounds [10]. Assessment of the impact of antibiotic use in chronic wounds will be crucial to establishing an effective, evidence-based regimen and to minimize inappropriate antibiotic use.

Traditionally, wound microbiota has been defined using culture-based methods; however, these methods are insufficient for characterizing complex polymicrobial communities, since many microbes cannot be cultured. There is an increasing number of chronic conditions and diseases associated with non-culturable or fastidious bacteria including bacterial vaginosis [11]; Whipple's disease [12]; and reactive arthritis [13]. Thus, it is critical to better define the role of fastidious and non-culturable bacteria in chronic wounds. Advanced molecular-based techniques, such as 16S rRNA gene-based pyrosequencing, can be used to characterize complex bacterial communities independent of culture-based enrichment. Preliminary 16S rRNA gene-based surveys of bacterial species associated with chronic wounds have found many putative wound colonizers that were not detected using standard culture-based methods and have revealed previously undescribed levels of bacterial diversity in chronic wounds [14]–[16]. Yet, these studies were mostly descriptive due to inherent limitations of standard statistical analyses against large, non-parametric community datasets. By applying community ecological analyses to evaluate the correlation between host and clinical factors and the chronic wound bacterial microbiota, it is possible to explore the associations between non-culturable bacteria with wound pathogenesis, chronicity, and infection.

In this study, we used 16S rRNA gene-based pyrosequencing analysis of the V3 region to characterize wound microbiota from a cohort of chronic wound patients and assessed the impact of diabetes and antibiotic therapy on bacterial communities. We have chosen the V3 region for its demonstrated ability to resolve bacterial taxa and produce comparable results to full-length (V1-V9) 16S rRNA gene sequences in 98.93% and 97.99% at the bacterial family and genus level, respectively, against human gut microbiota data [17]. We found that wounds were colonized by a wide-range of bacterial taxa including fastidious anaerobic pathogens that were not observed by culture-based analyses. Using community ecological analyses, we found evidence supporting clinical observations that diabetics were more likely to be colonized with Streptococcaceae and determined that recent antibiotic use was associated with increased Pseudomonadaceae colonization.

## Results

### Study Population

The demographic and clinical characteristics of the 24 participants included in the current analyses are listed in Table 1. Thirty-two individual wound samples were collected from the 24 participants at different times during the study. Fourteen of the wound samples were collected from patients who had been treated with topical or systemic antibiotics within 2 weeks prior to sample collection. The antibiotics administered prior to sample collection are listed in Table 2. Only 3 of the 14 antibiotic-treated wound samples were collected from participants who entered the study without receiving systemic or topical antibiotics but were treated during the period of observation.

**Table 1:** Demographic and clinical characteristics of study participants.

Characteristics	Value
Age (SD)	57.2 (15.6)
Sex	
Male	N = 10 (41.6%)
Female	N = 14 (58.4%)
Race	
Black	N = 11 (46.0%)
Caucasian	N = 13 (54.0%)
Primary Diagnosis (Wound Type)	
Decubitus	N = 7
Neuropathic	N = 7
Venous Stasis	N = 3
Post-Surgical	N = 3
Other	N = 4
Diabetes Mellitus	N = 12 (50.0%)
Antibiotic (for wound samples, N = 32)	
* Topical*	
24 h	N = 5 (15.6%)
* Systemic*	
24 h	N = 5 (15.6%)
2 weeks	N = 10 (31.3%)
Any antibiotic use in past 2 weeks	N = 14 (43.8%)

**Table 2:** Systemic antibiotics used within two weeks of sample collection.

Wound	Antibiotic
TG03	Unknown*
WS06	Keflex
WS08	Levofloxacin 750
WS10	Doxycycline
WS18	Bactrim DS, Flagyl
WS19	Clindamycin
WS20	Bactrim, Clindamycin
WS26	Levofloxacin
WS27	Clindamycin
WS30	Vantin, Flagyl
WS31	Bactrim
WS32	Bactrim DS, Flagyl
WS36	Cipro
WS38	Bactrim DS, Clindamycin
WS39	Bactrim DS

* The patient reported antibiotic use within the previous two weeks, but did not know the name of the antibiotic.

### Diverse Bacterial Communities Revealed by 16S rRNA gene-based Pyrosequencing Analyses

Bacterial taxonomic richness and diversity varied greatly among wounds examined in this study. Community richness and diversity were presented using Rarefaction and Shannon-Weaver Index plots, both of which provided insights into the structure and complexity of individual wound communities.

**Figure 1:**
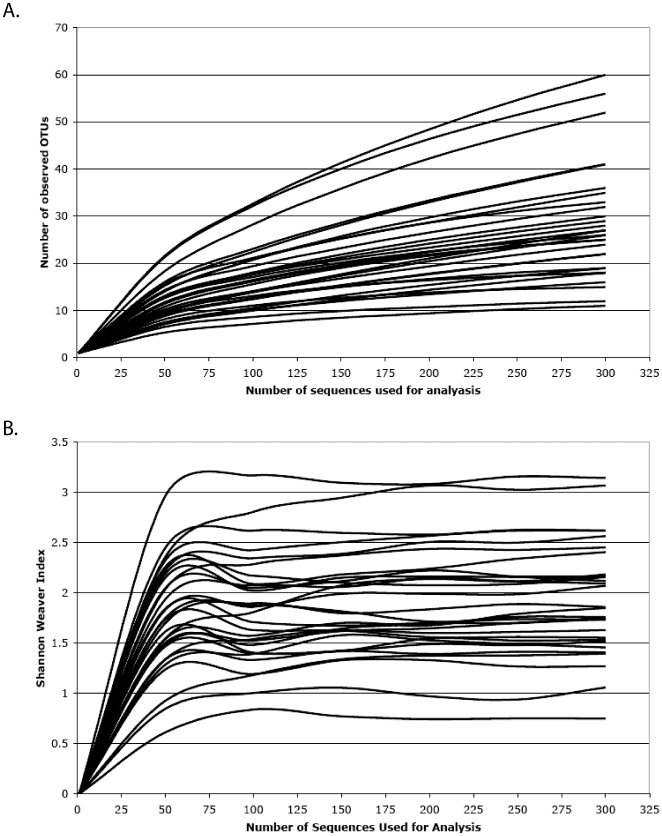
Rarefaction and Shannon Weaver index analyses were performed for each wound specimen. (A) Rarefaction curves were used to estimate richness (i.e., number of unique bacterial taxa) among samples. (B) Shannon Weaver Index curves were used estimate diversity (i.e., a combined assessment of the number of unique bacterial taxa and their abundance) among samples.

Rarefaction plots (Figure 1A) appear as two-component functions with a rapid increase in bacterial taxa observed until ∼50 sequences are sampled; after which, a second component with a lesser slope occurs in all cases. The first component includes the higher-frequency taxa that dominate the wound, while the second component represents less-frequent taxa. In individual wounds, the high-frequency taxa are as few as six or as great 25, with an average of ∼10. Less-frequent taxa more than double the observed taxonomic richness but not until 250 to 300 sequences have been analyzed.

Diversity value plots (Figure 1B) are driven entirely by the high-frequency taxa. Similar to the rarefaction plots, the diversity values increase until ∼50 sequences have been sampled. After which, the values stabilize with little change even when 300 sequences are sampled. This diversity index is based upon both the number of taxa and their frequency in the community; with numerous rare taxa having little effect on the final value. From these data, it is clear that 300 sequences are sufficient to estimate the bacterial community diversity values in individual wounds.

Bacterial community structure is determined through a mixture of high-frequency and low-frequency taxa, which are both potentially important to wound ecology and healing. Why some wounds have richer and more diverse communities is not apparent in these data and could be due to host, environmental or even stochastic processes. No trends in bacterial taxa richness or diversity values were evident among the diabetic or antimicrobial therapy groups (data not presented), though larger studies might be needed to detect these effects.

Taxonomic assignments were made with a bootstrap confidence range at ≥95% using the RDP Naïve Bayesian Classifier. The level of taxonomic resolution varied among sequence types identified in this study. While nearly all (98.8%) of the sequences analyzed were identified to the phylum level, the proportion of sequences successfully assigned to lower taxonomic groups decreased to 97.5% at the class level, 95.4% at the order level, 93.2% at the family level and then precipitously to 72.6% at the genus level. Importantly, only 53% of the Proteobacteria identified in this study were classified to the genus level.

**Figure 2:**
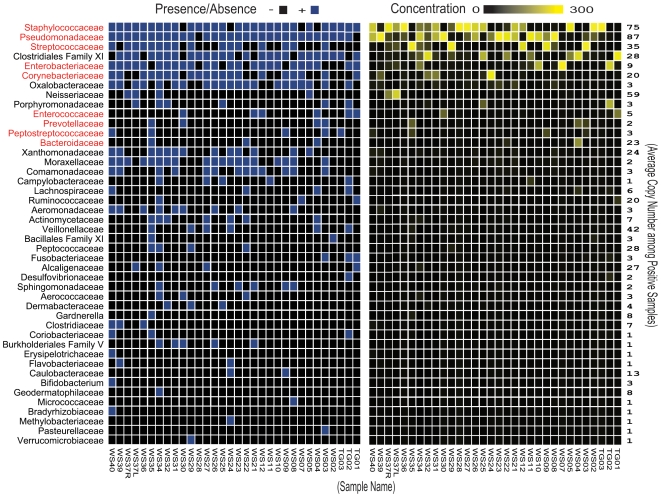
Heat map analysis of the 44 bacterial families detected using 16S rRNA gene-based pyrosequencing among chronic wound samples. The families in red are those that were successfully cultured at least once during the study. The presence/absence plot on the left shows the bacteria present in each of the wound samples. The abundance plot on the right shows the number of 16S rRNA gene pyrosequences (300 maximum) in each of the wound samples. The average copy number per positive sample for each detected bacterial family is shown on the far right. Many rare bacterial families are only visible on the presence/absence plot on the left.

Compared to culture-based analyses, 16S rRNA gene-based analyses revealed greater complexity at each taxonomic level (Table 3), identifying 44 bacterial families among the 32 wound samples (Figure 2). Most families were rare among samples and in low abundance when detected, thus confirming Shannon-Weaver Index analyses.

**Table 3:** Estimated complexity at different taxonomic levels by pyrosequencing and culture.

	Pyrosequencing			Culture		
Taxonomiclevel	Total # Among Samples	Range	Mean (SD)	Total # Among Samples	Range	Mean (SD)
Phylum	6	2–5	3.3 (0.9)	4	1–4	1.9 (0.9)
Class	13	3–9	5.4 (1.4)	5	1–4	1.9 (0.9)
Order	23	3–13	7.8 (2.4)	7	1–5	2.5 (1.2)
Family	44	3–22	10.0 (3.9)	9	1–5	2.5 (1.2)
Genera	58	3–24	9.4 (4.6)	14	1–6	2.7 (1.5)

Only nine out of the 44 bacterial families identified by 16S rRNA gene-based analyses were also successfully cultured in this study (in red, Figure 2). Many of the 35 discordant families (those detected by 16S rRNA gene-based analyses, but not by culture-based analyses) were relatively rare among samples; however, one family—Clostridiales family XI—was both prevalent (present in 78% of the samples) and abundant (>10% of sequences on average in positive samples). Five genera belonging to this family were identified among the wound samples including: *Anaerococcus*, *Finegoldia*, *Helcococcus*, *Parvimonas*, and *Peptoniphilus*.

Percent agreement between 16S rRNA gene-based and culture-based analyses averaged 71% for bacterial families that were cultured at least once (Table 4). 16S rRNA gene-based analyses were consistently equal to or more sensitive than culture-based methods at detecting these nine bacterial families. Culture-positive/pyrosequencing-negative discordance was rare; thus, most of the disagreement was due to culture-negative/pyrosequencing-positive discordance.

**Table 4:** Comparison between pyrosequencing and culture for detecting the nine bacterial families that were successfully cultured at least once, among all wound samples (n = 32).

	Pyro (−)	Pyro (+)	Total	Percent Agreement
**Bacteroidaceae**				93.75
Culture (−)	28	1	29	
Culture (+)	1	2	3	
Total	29	3	32	
**Corynebacteriaceae**				50.00
Culture (−)	10	14	24	
Culture (+)	2	6	8	
Total	12	20	32	
**Enterobacteriaceae**				56.25
Culture (−)	7	13	20	
Culture (+)	1	11	12	
Total	8	24	32	
**Enterococcaceae**				87.50
Culture (−)	24	2	26	
Culture (+)	2	4	6	
Total	26	6	32	
**Peptostreptococcaceae**				78.13
Culture (−)	25	5	30	
Culture (+)	2	0	2	
Total	27	5	32	
**Prevotellaceae**				81.25
Culture (−)	25	3	28	
Culture (+)	3	1	4	
Total	28	4	32	
**Pseudomonadaceae**				31.25
Culture (−)	1	21	22	
Culture (+)	1	9	10	
Total	2	30	32	
**Staphylococcaceae**				75.00
Culture (−)	3	7	10	
Culture (+)	1	21	22	
Total	4	28	32	
**Streptococcaceae**				87.50
Culture (−)	16	4	20	
Culture (+)	0	12	12	
Total	16	16	32	

### Antibiotic Therapy and Wound Microbiota

We compared bacterial communities in wounds from patients recently treated with antibiotics to those from untreated patients using non-metric multidimensional scaling (nMDS) and multiresponse permutation procedure (MRPP). We further identified the bacterial families that best distinguish the two antibiotic use groups using Dufrêne & Legendre indicator analysis.

**Figure 3:**
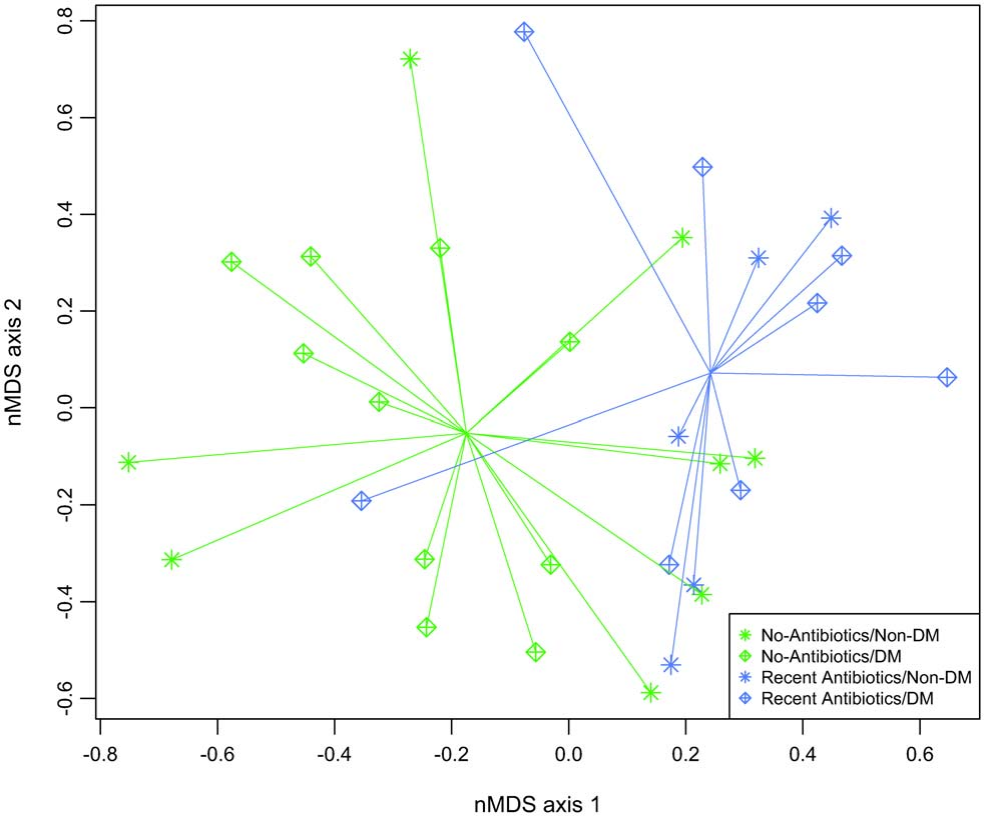
The nMDS ordination plot comparing wound bacterial communities from antibiotic treated participants and untreated participants. Each data point in nMDS plot represent the bacterial community identified from a single wound specimen. Comparison using MRPP found that the antibiotic treated and untreated wound microbiota are significantly different (*p* = 0.0069).

The nMDS and MRPP analyses showed significantly different bacterial communities in patients that were untreated or treated with antibiotic in the two weeks prior to sample collection (Figure 3). The indicator analyses found that recent antibiotic use was associated with increased abundance of Corynebacteriaceae, Oxalobacteraceae, and Pseudomonadaceae.

We compared the prevalence of Corynebacteriaceae, Oxalobacteraceae, and Pseudomonadaceae between antibiotic use groups by calculating the mean difference in sequence counts and performing univariate pair-wise comparisons using a t-test statistic and Monte Carlo-based re-sampling. We found a large proportional increase in each of these indicator taxa in the antibiotic treated group, but the within-group variances were also large (Table 5). Univariate comparisons showed that Pseudomonadaceae was significantly higher in wounds from antibiotic treated participants compared to untreated participants. The increases in Corynebacteriaceae and Oxalobacteraceae were not significant after the Bonferroni correction (Table 5). To further evaluate the association between Pseudomonadaceae and antibiotic use, we examined the prevalence data in three participants from whom the pre- and post-antibiotic data were available. In this small subset, we found a significant increase in Pseudomonadaceae after antibiotic therapy (∼27 fold average increase; *p* = 0.047).

**Table 5:** Comparison of indicator species prevalence (out of n = 300 sequences for each sample) between untreated and antibiotic treated wounds.

	No Recent ABx	Recent ABx		
Taxonomic Group	Mean (SD)	Mean (SD)	Δ Mean	Empirical p-value*
*Corynebacteriaceae*	2.5 (3.6)	25.4 (47.1)	22.9	0.019
*Oxalobacteraceae*	2.9 (6.3)	9.1 (8.8)	6.2	0.020
*Pseudomonadaceae*	39.9 (51.0)	110.1 (75.2)	70.2	0.0046**

* The empirical p-values comparing the prevalence of indicator species between the two antibiotic use groups were generated using the Monte Carlo method. The statistical significance level after the Bonferroni correction was 0.05/3 = 0.017.

** The increase in *Pseudomonadaceae* in the antibiotic treated group was significant at *p* = 0.0046<0.017.

### Diabetes and Wound Microbiota

We further evaluated the association between diabetes and chronic wound microbiota using nMDS, MRPP, indicator analysis, and indicator prevalence as described above. The nMDS and MRPP analyses did not reveal a significant difference between the diabetic and non-diabetic wound microbiota on the community level (data not shown). Indicator analysis showed that differences in Streptococcaceae prevalence best distinguished the diabetic from non-diabetic wound microbiota. Prevalence comparisons of Streptococcaceae in diabetic wounds (Mean = 49.83, SD = 69.97) versus non-diabetic wounds (Mean = 3.92, SD = 11.54) showed large between-group and within-group variances. The difference in Streptococcaceae prevalence between diabetic and non-diabetic wounds was significant (*p* = 0.015) using the Monte Carlo method.

### Interaction Between Diabetes And Antibiotic Use On The Wound Microbiota

At the genus level, indicator analysis showed that *Streptococcus* was a shared indicator in antibiotic use and diabetes, with a decreased prevalence of *Streptococcus* in antibiotic treated wounds compared to untreated wounds and an increased prevalence of *Streptococcus* in diabetic wounds compared to non-diabetic wounds (data not shown).

We assessed the presence of interaction between diabetes and antibiotic use on the community-scale using nMDS and MRPP, which revealed a significant interaction between diabetes and antibiotic use (data not shown).

We assessed interaction between diabetes and antibiotic use in *Streptococcus* colonization using multiple logistic regression. Antibiotic use was associated with a 41% reduction in risk of *Streptococcus* colonization in diabetics (*p* = 0.009) but no significant risk reduction in non-diabetics (*p* = 0.21). Additionally, in those not recently treated with antibiotics, the diabetic wounds were 63 times more likely to be colonized with *Streptococcus* than the non-diabetic wounds (OR = 63, 95% CI = 3.32, 1194).

## Discussion

Modern molecular tools such as 16S rRNA gene-based pyrosequencing provide powerful means to define chronic wound bacteria. We found that chronic wounds supported complex microbial communities comprised of a wide-range of bacterial taxa including fastidious anaerobic bacteria that were not observed using culture-based methods. The bacterial wound communities characterized in this study were similar in composition to those reported by other groups using 16S rRNA gene-based methods [14], [16]. The number and proportion of bacterial taxa ranged greatly in individual wounds. Additional research involving longitudinal sampling is needed to understand the dynamics of bacterial communities in chronic wounds.

Not surprisingly, bacterial diversity was substantially higher when determined by 16S rRNA gene-based pyrosequencing analysis as compared to the culture-based analyses. The limitations of culture-based methods to characterize diverse bacterial communities from environmental and clinical samples have been noted previously; however, many organisms missed by culture-based methods in the current study were theoretically culturable using conventional methods. Some of the organisms that were missed by culture-based methods were proportionally rare and may have been masked by more dominant organisms in the culture media. Other organisms, such as those belonging to the Neisseriaceae and Campylobacteriaceae families, are fastidious and thus require special culture media that are not typically used when culturing wounds in clinical laboratories. Obligate anaerobes, such as Clostridiales family XI, are particularly difficult to grow and were not identified using culture-based methods in the current study. Using 16S rRNA gene-based sequence analysis, we identified bacteria from Clostridiales family XI in 25 of the 32 wounds analyzed. Five genera from this family were identified: *Anaerococcus*, *Finegoldia*, *Helcococcus*, *Parvimonas*, and *Peptoniphilus*. Complex anaerobic microbiota that include the Clostridiales family XI have been associated with diseases such as bacterial vaginosis [18], [19], diabetic foot ulcers [16], [20], necrotizing fasciitis [21], and periodontal disease [22], [23]. Thus, the data presented here highlight the limitations of routine clinical culture to detect potentially important fastidious pathogens.

We compared 16S rRNA gene-based pyrosequencing to culture-based analyses for detecting bacterial taxa that were cultured at least once during the study. In this analysis, we found that detection of these culturable bacteria was consistently greater by 16S rRNA gene-based pyrosequencing as compared to culture-based methods. While culture-negative/pyrosequencing-positive discordant pairs were common, culture-positive/pyrosequencing-negative discordant pairs were rare. All bacterial families identified by culture-based methods were targeted by the amplification primers used in the current study; therefore, insufficient sampling is the most likely explanation for the rare culture-positive/pyrosequencing-negative discordant pairs [24]. In contrast, there are several possible explanations for culture-negative/pyrosequencing-positive discordant pairs, including: 1) *Molecular detection of viable/non-culturable bacteria.* Viable/non-culturable bacteria may include non-planktonic bacteria existing in biofilms, which are common in chronic wounds; 2) *Molecular detection of bacteria that were proportionately rare within the community and masked by more dominant bacteria in culture media.* This is an expected limitation of using non-selective culture media; 3) *Molecular detection of DNA from dead (non-viable) bacteria.* Detecting DNA from dead bacteria is a common criticism of using DNA-based molecular methods to characterize microbial communities. While we acknowledge this potential bias, our data suggest that underestimation by culture-based methods is far more likely. Strategies to reduce or eliminate nucleic acids from dead bacteria, such as incorporation of DNA digestion steps prior to cell lysis, may help minimize detection of dead bacteria. Another approach would be to perform RNA (cDNA)-based analysis, which would identify bacteria that are metabolically active.

The V3 hypervariable region is one of the most phylogenetically informative regions of the 16S gene, but this study illustrates the limitations of this region for taxonomic assignment using the RDP classifier. Previous work with the RDP classifier indicated that 83.2% of the bacteria in the Bergey corpus could be accurately assigned to the appropriate genus using 200 base segments of the 16S gene. Three phyla—Firmicutes, Proteobacteria, and Actinobacteria—were the most commonly misclassified [25]. The 16S sequences used in the current study were all greater than or equal to 200 bases, with most (89.1%) of the sequences falling into Firmicutes, Proteobacteria, and Actinobacteria phyla. High percentages of the Firmicutes and Actinobacteria sequences were successfully assigned to the genus level, 88.0% and 82.3%, respectively; however, only 53.0% of the Proteobacteria sequences were successfully assigned to the genus level. Most of the Proteobacteria sequences belonged to four families: Pseudomonadaceae, Enterobacteriaceae, Oxalobacteraceae and Neisseriaceae. Varying proportions of all four families were successfully assigned to the genus level: 69.2%, 29.0%, 23.8% and 1.8%, respectively. A combination of sequence homoplasy and inaccurate database assignments (i.e., falsely assigned reference sequences in the RDP database) could have contributed to our inability to assign Proteobacteria sequences to taxonomic groups below the family level.

Recent antibiotic use was associated with increased Pseudomonadaceae colonization in the current study. A similar association was reported previously in a study of tracheal colonization among critically ill, intubated patients [26]. In this earlier study, daily endotracheal aspirates were collected from patients after intubation. Bacterial communities from six of the seven patients shifted from relatively diverse communities to *Pseudomonas*-dominated communities with the administration of antibiotics. Interestingly, *Pseudomonas* isolates collected from these patients were susceptible to the administered antibiotics in laboratory drug-susceptibility tests. It was hypothesized that this paradoxical finding was the result of differential susceptibility of *Pseudomonas* growing planktonically versus those growing in biofilms [26]. Biofilms are thought to be an important factor contributing to the chronicity of certain non-healing wounds. Administration of antibiotics may select for biofilm-producing organisms such as *Pseudomonas* and delay rather than aid wound healing.

One of the limitations of our study was its observational design, which may have resulted in selection bias. Participants were not excluded from the study based on prior therapies and eight patients entered the study having been treated with antibiotics within the previous two weeks. Those participants entering the study with recent exposure to antibiotics may have been treated in response to pre-existing *Pseudomonas* colonization or infection, which may have biased the observed association between antibiotic use and increased *Pseudomonas* colonization. Three participants entered the study without recent exposure to antibiotics and were treated during the study. Sub-analyses of these three participants revealed a significant increase in *Pseudomonas* abundance after antibiotic treatment. These data support the hypothesis that antibiotic use selected for increased *Pseudomonas* colonization, but additional prospective studies will have to be conducted to confirm these findings.

The 16S rRNA gene-based pyrosequencing analysis confirmed our clinical observations indicating that diabetics were significantly more likely to be colonized with *Streptococcus*. Increased *Streptococcus* colonization may be an important factor contributing to the disproportionate morbidity associated with chronic wounds among diabetics compared to non-diabetics [27]. Antibiotic use was associated with decreased *Streptococcus* colonization among diabetics and thus may be a suitable therapeutic option for treating diabetic patients with *Streptococcus* infections. Further studies are needed to confirm the association between diabetes and *Streptococcus* colonization and to elucidate the biological basis for this association.

Currently, wound management is largely empirical and based on principles of reducing bacterial load and preventing infection [28]; however, the complexity of the wound environment makes it likely that antimicrobial therapy could result in unintended consequences. We have little prospective data on the microbiological response to antimicrobial wound therapies. Thus, application of 16S rRNA gene-based pyrosequencing to characterize wound microbial communities with respect to clinical outcomes and therapeutic interventions (particularly antibiotic treatments) will provide critical insights into the roles of microbiota in wound healing and the impacts of wound therapies.

## Materials and Methods

### Clinical specimens

The study was approved by the Johns Hopkins Institutional Review Board. Chronic wound tissue samples were collected from 24 patients attending the Johns Hopkins Wound Center, a tertiary wound center in Baltimore, MD. After consent and local anesthesia, tissue was collected from the wound base with a 3 mm curette. Tissue samples were evaluated by qualitative aerobic and anaerobic culture in a CLIA-certified laboratory as follows: An unweighed portion of the sample was homogenized in a 15 ml conical tube using a sterile swab. One drop of the homogenate was plated on selective and non-selective media including sheep blood agar, MacConkey and chocolate agar plates and grown aerobically at 37°C for 24 hours. Columbia agar (CNA), CDC agar, and BBE/LKV were used for the recovery of anaerobic organisms. Plates for anaerobic assessment were incubated in the BD anaerobic gas pouch system at 35°C for 4–7 days.

### DNA isolation from wound tissue samples

Genomic DNA was extracted from wound samples using a bead-beating and enzymatic lysis protocol, followed by purification using a QIAamp DNA Mini Kit (Qiagen, USA). Briefly, frozen wound samples (10 to 100 mg) were thawed on ice then suspended in 0.5 ml of TE50 (10 mM Tris-HCl +50 mM EDTA, pH 8.0) solution and allowed to soak on ice for 5 min before being vortexed. The suspension was transferred to a clean, sterile bead-beating tube (MP Biomedicals, USA) and kept on ice. A lytic enzyme cocktail was prepared at the time of extraction and added to each sample as follows: 50 µl Lysozyme (450 kU ml^−1^), 6 µl Mutanolysin (25 kU ml^−1^), 3 µl Lysostaphin (4 kU ml^−1^) and 41 µl TE50 for a final volume of 100 µl per sample. Samples were digested by incubating at 37°C for 60 min in a dry heat block before centrifugation at 1200 rpm for 1 min. To each digested sample, 750 mg of sterile 0.1 mm diameter zirconia silica beads (BioSpec, Products Inc. USA) were added. Bead-beating was performed for 1 min at 2100 rpm using a BioSpec Mini-Bead Beater-96. Following bead disruption, the tubes were centrifuged at 1200 rpm for 1 min. A total of 200 µl of crude lysate was transferred to a new, sterile microcentrifuge tube. To each tube, 25 µl of Proteinase K (20 mg/ml (>600 mAU/ml)) and 200 µl of Qiagen buffer AL were added. Samples were mixed by pulse-vortexing for 15 sec and then incubated at 56°C for 10 min before being centrifuged at 1200 rpm for 1 min. For each 200 µl crude lysate, 20 µl of 3 M sodium acetate, pH 5.5 was added followed by 200 µl of molecular grade ethanol (96–99.5%). Vortexing was repeated for an additional 15 sec before being centrifuged at 1200 rpm for 1 min. From this point onward, purification was carried out using the QIAmp DNA Purification from Blood or Body Fluids (Vacuum Protocol) as per manufacturer's instructions. Purified genomic DNA was stored at −80°C until analysis.

### Pyrosequencing library synthesis for parallel tagged sequencing on the 454® platform

The 16S rRNA gene was amplified in two replicate 50 µl reaction volumes. In each 50 µl reaction, 3 µl was added to 47 µl of PCR reaction mix containing 450 nM of each broad range forward (5′-CCTACGGGAGGCAGCAGT-3′) and reverse primer (5′-GGACTACCAGGGTATCTAATCCTGTT-3′) [29], 1X PCR buffer without MgCl_2_ (Invitrogen), 3 mM MgCl_2_, 0.2 mM dNTP mix, 0.02 U platinum *Taq* (Invitrogen) using the following touch-down PCR condition: 90 s at 95°C for initial denaturation, 30 s at 95°C for denaturation, 30 s at 64°C for annealing, 30 s at 72°C for extension with the annealing temperature decreasing by 0.3°C for each subsequent cycle for 34 cycles, followed by 5 min at 72°C for final extension. Subsequent purification, blunt-end repair, adapter ligation, amplicon quantification and pooling, restriction digestion, and pyrosequencing library generation were carried according to a previously published protocol [30]. The sample-specific, palindromic, self-hybridizing barcodes used in the tagging reactions were generated using a self-complementary 8-nt barcode and a rare restriction site according the same protocol.

### Pyrosequencing using the 454® platform

The pooled tagged single-stranded pyrosequencing library underwent fusion PCR and pyrosequencing using a Roche 454 FLX Pyrosequencer (Roche Life Sciences, USA) according to the manufacturer instructions [31] at the Institute for Genome Sciences, Genomic Resource Center.

### Sequence processing

Experimental sequences were processed using a custom PERL script, which performed the following: the script filtered the sequence files and retained only sequences that were 200-nt or longer. It then searched for a single barcode sequence in each FASTA sequence, binned each sequence accordingly, and scanned each binned sequence for the 16S forward primer sequence. The script then trimmed off the forward primer sequence and oriented the remaining sequence such that all sequences begin with the 5′ end according to standard sense strand conventions. As a result of our processing, sequences that were shorter than 200-nt or had multiple barcode or primer motifs were excluded from the analysis. We included only sequences with the forward primer motif to ensure that the highly informative V3 region was available for taxonomic assignment. The trimmed sequences from each barcode bin were aligned using the NAST alignment tool (http://greengenes.lbl.gov) [32]. After alignment, the number of sequences examined per wound sample was equilibrized to 300 sequences by sampling randomly without replacement to facilitate subsequent taxa abundance analyses. Samples with fewer than 300 sequences were excluded. The cutoff of n = 300 was established based on richness (rarefaction) and diversity (Shannon-Weaver Index) analyses using DOTUR [34], which indicated that samples were sufficiently sampled after ≥300 sequences.

### Taxonomic assignment

Unaligned, sequences in the equilibrated dataset were given taxonomic assignments at a bootstrap confidence range of ≥95% using the Ribosomal Database Project's Naïve Bayesian Classifier tool (RDP classifier) [25], [35].

### Rarefaction and diversity analyses

Distance matrices based on taxa abundance were generated with the Dnadist tool of PHYLIP 3.67 using the default settings [33]. Rarefaction and Shannon Weaver index estimations were determined by DOTUR [34] and plotted in Microsoft Excel (Microsoft Corp., USA).

### Statistical analyses

All statistical analyses were performed using our equilibrated dataset (n = 300 sequences per sample). Community-scale multivariate analyses including non-metric multidimensional scaling (nMDS), multiresponse permutation procedure (MRPP), and the Dufrêne & Legendre indicator analyses were performed in R [36] using statistical packages vegan [37], ecodist [38], BiodiversityR [39], and labdsv [40]. The nMDS analysis is a nonparametric ordination-based method for reducing ecological community data complexity and identifying meaningful relationships amongst communities, while the MRPP analysis is another nonparametric method for testing the null hypothesis of no-difference between communities by comparing the experimental with the expected within-group difference through an iterative randomization process. The indicator species analysis further identifies the bacterial taxa that are significantly unique to each environment (e.g., clinical variables of interest). The nonparametric nature of these ecological analysis methods is highly suitable for human bacterial community data, which are frequently zero-rich, highly-skewed, and non-normal and remains non-normally distributed post-data transformation. Significance level for MRPP and the Dufrêne & Legendre indicator analyses were set at α = 0.05.

Comparative analysis of mean indicator prevlaence between environments (e.g. diabetics versus non-diabetics) were also performed in R using custom codes. Briefly, using the taxa abundance-based distance matrices, a t-statistic was calculated and the underlying null distribution was estimated using Monte-Carlo based resampling (n = 10,000 permutations). A two-tailed empirical p-value was generated by comparing the unpermuted data with the estimated null distribution. Significance levels were set at α = 0.05 with the appropriate Bonferroni correction (α/n), with n =  number of tests performed for a single environment.

Assessment for the interaction between antibiotic use and diabetes and the percent agreements in the comparison of 16S rRNA gene-based and culture-based results were performed using multivariate logistic regression and the kappa-statistic, respectively in STATA 9 (StataCorp, USA).

## References

[1] Bowler PG (2002). Wound pathophysiology, infection and therapeutic options.. Ann Med.

[2] Bowler PG, Duerden BI, Armstrong DG (2001). Wound microbiology and associated approaches to wound management.. Clin Microbiol Rev.

[3] Mustoe TA, O'Shaughnessy K, Kloeters O (2006). Chronic wound pathogenesis and current treatment strategies: A unifying hypothesis.. Plast Reconstr Surg.

[4] Edwards R, Harding KG (2004). Bacteria and wound healing.. Curr Opin Infect Dis.

[5] Wysocki AB (2002). Evaluating and managing open skin wounds: Colonization versus infection.. AACN Clin Issues.

[6] Howell-Jones RS, Wilson MJ, Hill KE, Howard AJ, Price PE (2005). A review of the microbiology, antibiotic usage and resistance in chronic skin wounds.. J Antimicrob Chemother.

[7] O'Meara S, Al-Kurdi D, Ovington LG (2008). Antibiotics and antiseptics for venous leg ulcers.. Cochrane Database Syst Rev.

[8] O'Meara S, Cullum N, Majid M, Sheldon T (2000). Systematic reviews of wound care management: (3) antimicrobial agents for chronic wounds; (4) diabetic foot ulceration.. Health Technol Assess.

[9] Howell-Jones RS, Price PE, Howard AJ, Thomas DW (2006). Antibiotic prescribing for chronic skin wounds in primary care.. Wound Repair Regen.

[10] Lipsky BA (2008). New developments in diagnosing and treating diabetic foot infections.. Diabetes Metab Res Rev.

[11] Davies CE, Wilson MJ, Hill KE, Stephens P, Hill CM (2001). Use of molecular techniques to study microbial diversity in the skin: Chronic wounds reevaluated.. Wound Repair Regen.

[12] Schneider T, Moos V, Loddenkemper C, Marth T, Fenollar F (2008). Whipple's disease: New aspects of pathogenesis and treatment.. Lancet Infect Dis.

[13] Rihl M, Klos A, Kohler L, Kuipers JG (2006). Infection and musculoskeletal conditions: Reactive arthritis.. Best Pract Res Clin Rheumatol.

[14] Andersen A, Hill KE, Stephens P, Thomas DW, Jorgensen B (2007). Bacterial profiling using skin grafting, standard culture and molecular bacteriological methods.. J Wound Care.

[15] Dowd SE, Sun Y, Secor PR, Rhoads DD, Wolcott BM (2008). Survey of bacterial diversity in chronic wounds using pyrosequencing, DGGE, and full ribosome shotgun sequencing.. BMC Microbiol.

[16] Dowd SE, Wolcott RD, Sun Y, McKeehan T, Smith E (2008). Polymicrobial nature of chronic diabetic foot ulcer biofilm infections determined using bacterial tag encoded FLX amplicon pyrosequencing (bTEFAP).. PLoS ONE.

[17] Huse SM, Dethlefsen L, Huber JA, Welch DM, Relman DA (2008). Exploring microbial diversity and taxonomy using SSU rRNA hypervariable tag sequencing.. PLoS Genet.

[18] Spear GT, Sikaroodi M, Zariffard MR, Landay AL, French AL (2008). Comparison of the diversity of the vaginal microbiota in HIV-infected and HIV-uninfected women with or without bacterial vaginosis.. J Infect Dis.

[19] Marrazzo JM, Thomas KK, Fiedler TL, Ringwood K, Fredricks DN (2008). Relationship of specific vaginal bacteria and bacterial vaginosis treatment failure in women who have sex with women.. Ann Intern Med.

[20] Goldstein EJ, Citron DM, Merriam CV, Warren Y, Tyrrell KL (2002). General microbiology and in vitro susceptibility of anaerobes isolated from complicated skin and skin-structure infections in patients enrolled in a comparative trial of ertapenem versus piperacillin-tazobactam.. Clin Infect Dis.

[21] Lee S, Roh KH, Kim CK, Yong D, Choi JY (2008). A case of necrotizing fasciitis due to streptococcus agalactiae, arcanobacterium haemolyticum, and finegoldia magna in a dog-bitten patient with diabetes.. Korean J Lab Med.

[22] Siqueira JF, Rocas IN (2008). Clinical implications and microbiology of bacterial persistence after treatment procedures.. J Endod.

[23] Rocas IN, Siqueira JF (2008). Root canal microbiota of teeth with chronic apical periodontitis.. J Clin Microbiol.

[24] DiGiulio DB, Romero R, Amogan HP, Kusanovic JP, Bik EM (2008). Microbial prevalence, diversity and abundance in amniotic fluid during preterm labor: A molecular and culture-based investigation.. PLoS ONE.

[25] Wang Q, Garrity GM, Tiedje JM, Cole JR (2007). Naive bayesian classifier for rapid assignment of rRNA sequences into the new bacterial taxonomy.. Appl Environ Microbiol.

[26] Flanagan JL, Brodie EL, Weng L, Lynch SV, Garcia O (2007). Loss of bacterial diversity during antibiotic treatment of intubated patients colonized with pseudomonas aeruginosa.. J Clin Microbiol.

[27] Greenhalgh DG (2003). Wound healing and diabetes mellitus.. Clin Plast Surg.

[28] Fonder MA, Lazarus GS, Cowan DA, Aronson-Cook B, Kohli AR (2008). Treating the chronic wound: A practical approach to the care of nonhealing wounds and wound care dressings.. J Am Acad Dermatol.

[29] Nadkarni MA, Martin FE, Jacques NA, Hunter N (2002). Determination of bacterial load by real-time PCR using a broad-range (universal) probe and primers set.. Microbiology.

[30] Meyer M, Stenzel U, Hofreiter M (2008). Parallel tagged sequencing on the 454 platform.. Nat Protoc.

[31] McKenna P, Hoffmann C, Minkah N, Aye PP, Lackner A (2008). The macaque gut microbiome in health, lentiviral infection, and chronic enterocolitis.. PLoS Pathog.

[32] DeSantis TZ, Hugenholtz P, Keller K, Brodie EL, Larsen N (2006). NAST: A multiple sequence alignment server for comparative analysis of 16S rRNA genes.. Nucleic Acids Res.

[33] Felsenstein J (1989). PHYLIP—phylogeny inference package (version 3.2).. Cladistics.

[34] Schloss PD, Handelsman J (2005). Introducing DOTUR, a computer program for defining operational taxonomic units and estimating species richness.. Appl Environ Microbiol.

[35] Cole JR, Wang Q, Cardenas E, Fish J, Chai B (2009). The ribosomal database project: Improved alignments and new tools for rRNA analysis.. Nucleic Acids Res.

[36] R Development Core Team (2008). R: A language and environment for statistical computing..

[37] Oksanen J, Kindt R, Legendre P, O'Hara B, Simpson GL (2009). vegan: Community Ecology Package.. http://CRAN.R-project.org/package=vegan.

[38] Goslee SC, Urban DL (2007). The ecodist package for dissimilarity-based analysis of ecological data.. Journal of Statistical Software.

[39] Kindt R, Coe R (2005). Tree diversity analysis. A manual and software for common statistical methods for ecological and biodiversity studies..

[40] Roberts DW (2007). labdsv: Ordination and Multivariate Analysis for Ecology.. http://ecology.msu.montana.edu/labdsv/R.

